# Tumor-derived CXCL8 signaling augments stroma-derived CCL2-promoted proliferation and CXCL12-mediated invasion of PTEN-deficient prostate cancer cells

**DOI:** 10.18632/oncotarget.2052

**Published:** 2014-06-01

**Authors:** Pamela J. Maxwell, Jessica Neisen, Johanna Messenger, David J.J. Waugh

**Affiliations:** ^1^ Centre for Cancer Research and Cell Biology, Queen's University Belfast, Belfast, Northern Ireland

**Keywords:** CXCL8, CCL2, CXCL12, CXCR4, prostate cancer, PTEN

## Abstract

Impaired *PTEN* function is a genetic hallmark of aggressive prostate cancers (CaP) and is associated with increased CXCL8 expression and signaling. The current aim was to further characterize biological responses and mechanisms underpinning CXCL8-promoted progression of PTEN-depleted prostate cancer, focusing on characterizing the potential interplay between CXCL8 and other disease-promoting chemokines resident within the prostate tumor microenvironment. Autocrine CXCL8-stimulation (i) increased expression of CXCR1 and CXCR2 in PTEN-deficient CaP cells suggesting a self-potentiating signaling axis and (ii) induced expression of CXCR4 and CCR2 in PTEN-wild-type and PTEN-depleted CaP cells. In contrast, paracrine CXCL8 signaling induced expression and secretion of the chemokines CCL2 and CXCL12 from prostate stromal WPMY-1 fibroblasts and monocytic macrophage-like THP-1 cells. *In vitro* studies demonstrated functional co-operation of tumor-derived CXCL8 with stromal-derived chemokines. CXCL12-induced migration of PC3 cells and CCL2-induced proliferation of prostate cancer cells were dependent upon intrinsic CXCL8 signaling within the prostate cancer cells. For example, in co-culture experiments, CXCL12/CXCR4 signaling but not CCL2/CCR2 signaling supported fibroblast-mediated migration of PC3 cells while CXCL12/CXCR4 and CCL2/CCR2 signaling underpinned monocyte-enhanced migration of PC3 cells. Combined inhibition of both CXCL8 and CXCL12 signaling was more effective in inhibiting fibroblast-promoted cell motility while repression of CXCL8 attenuated CCL2-promoted proliferation of prostate cancer cells. We conclude that tumor-derived CXCL8 signaling from PTEN-deficient tumor cells increases the sensitivity and responsiveness of CaP cells to stromal chemokines by concurrently upregulating receptor expression in cancer cells and inducing stromal chemokine synthesis. Combined chemokine targeting may be required to inhibit their multi-faceted actions in promoting the invasion and proliferation of aggressive CaP.

## INTRODUCTION

Large scale sequencing and molecular pathology analysis is providing deeper insight into the genetic underpinnings of prostate cancer, heightening anticipation of near-term and widespread access to precision-based therapy of prostate cancer [1]. One of the best characterized common genetic events in prostate cancer relates to the impaired function of *PTEN*, a haplo-insufficient tumor suppressor gene, whose primary cellular activity is to regulate the phosphatidylinositol-3-kinase (PI3K)/Akt signaling pathway. Allelic loss, epigenetic silencing and/or acquisition of functionally-inactivating mutations have all been shown to contribute to the impairment of *PTEN* in prostate cancer [2,3]. Elegant *in vivo* genetically-engineered mouse models have shown that heterozygous or homozygous deletion of *PTEN* in the prostate epithelium [4] or alternatively, constitutive activation of the downstream effector PKB/Akt [5] underpins the development of a prostate pathology recapitulating human prostatic intra-epithelial neoplasia (PIN), a pre-malignant condition. In further experimental models, the combination of PTEN loss with ERG over-expression or Tp53 mutation has been shown to promote the transition to invasive prostate carcinoma [6,7] while epidemiological studies conform the relevance of PTEN to aggressive prostate cancer [8]. In support of this, a recently published longitudinal molecular pathology analysis indicated that mutation of PTEN was associated with the lethal phenotype of prostate cancer [9]. Furthermore, other recent studies support that functional loss of *PTEN* is correlated with the relapse of prostate cancer after radical prostatectomy or radiotherapy [10,11]. Therefore, while pre-clinical and clinical evidence suggests that increased signaling of the PTEN/PI3K/Akt pathway is considered to be a sustaining drive in the development and progression of this disease, our understanding of the key biological mediators and microenvironment responses that underpin and define the more aggressive behavior of *PTEN*-depleted prostate cancer remains to be fully defined.

Chemokines are critical signaling mediators, driving communication between malignant epithelial cells and the surrounding microenvironment, and contribute to multiple hallmarks of cancer. Overexpression and increased activity of three specific chemokines termed CCL2, CXCL12 and CXCL8 have been strongly implicated in the progression of prostate cancer [12-14]. Increased expression and activity of CCL2 and CXCL12 has been reported in both the localized prostate cancer and within the microenvironment of bone metastases [13-18], while prior work from our own group has confirmed elevated CXCL8 expression in human prostate cancer tissue [19].

Our recent research has established an important contextual relationship of impaired PTEN function to the over-expression of CXCL8 in prostate tumor cells. Conversely, modulation of this tumor suppressor gene had no impact on the expression of either CCL2 or CXCL12 in prostate cancer cells [20]. Other studies report that expression of CCL2 and CXCL12 may originate from stromal cells and regulate tumor cell behavior through promotion of paracrine signaling [12,13,15,18,21]. Therefore, the initial objective of this study was to establish the existence of interplay between tumor-derived CXCL8 from PTEN-deficient prostate cancer cells and the potentiation of stromal-derived CCL2 and CXCL12 expression and/or signaling. Our further objective was to characterize the functional consequences of this interplay in the context of augmenting the proliferation and/or migration of PTEN-deficient prostate cancer cells. In this study, we report a co-operative role between CXCL8 and stromal-derived chemokines, establishing a multi-faceted chemokine cross-talk between PTEN-deficient tumor and stroma that sustains the proliferation, migration and invasion of these aggressive prostate cancer cells.

## RESULTS

### CXCL8 signaling regulates chemokine receptor expression in the prostate epithelium

**Figure 1:**
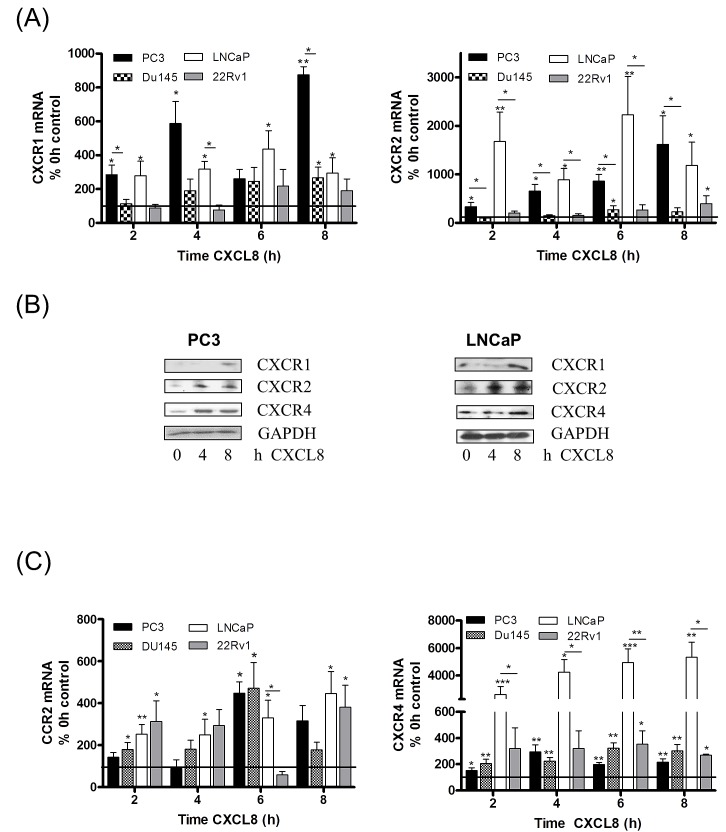
Autocrine CXCL8 signaling increases chemokine receptor expression in prostate cancer cells **(A)** Bar graph illustrating qPCR validation of CXCR1 (left panel) and CXCR2 (right panel) gene expression in multiple prostate cancer cell lines, subjected to stimulation with 3nM rh-CXCL8. **(B)** Immunoblots demonstrating increased protein levels of chemokine receptors in PC3 (left panel) and LNCaP cells (right panel) following exposure to 3nM rh-CXCL8. **(C)** Bar graph illustrating qPCR validation of CCR2 (left panel) and CXCR4 (right panel) gene expression in multiple prostate cancer cell lines, subjected to stimulation with 3nM rh-CXCL8. Data shown in (A) and (C) is the mean ± S.E.M value, determined from a minimum of 4 replicate experiments. Statically significant differences in expression were determined by performing a two-tailed Mann-Whitney U-test (*p<0.05; ** p<0.01; ***p<0.001).

Our prior studies demonstrated that silencing of PTEN expression increased the transcription and secretion of CXCL8 and increased gene expression of the receptors CXCR1 and CXCR2 in prostate cancer cells [20]. In the current study, our initial experiments sought to investigate how elevated levels of CXCL8 signaling may regulate chemokine receptor expression in the prostate epithelium. The ability of the recombinant-human CXCL8 (rh-CXCL8) stimulus to regulate chemokine receptor expression was studied in representative androgen-sensitive (LNCaP and 22Rv1) and castrate-resistant (PC3 and DU145) cell lines. QPCR analysis confirmed that exogenous rh-CXCL8 administration significantly increased CXCR1 (Fig 1A, left panel) and CXCR2 (Fig 1A, right panel) mRNA expression in the PTEN-null PC3 and LNCaP cells. No change in CXCR1 or CXCR2 mRNA expression was detected in PTEN-expressing DU145 or 22Rv1 cells (Fig 1A). The importance of PTEN in regulating this response was exemplified using isogenic lines. Administration of rh-CXCL8 induced CXCR1/2 mRNA expression in a PTEN-depleted clone (sh11.02) but not a PTEN-expressing (NT.01) clone of the DU145 cell line (Figure S1A). Furthermore, immunoblotting confirmed the CXCL8-stimulated increase in CXCR1 and CXCR2 receptor expression in PC3 and LNCaP cells (Fig 1B). Therefore, these initial experiments suggest that CXCL8 signaling can potentiate expression of the receptors transducing its biological activity in PTEN-deficient prostate carcinoma cells.

The effect of CXCL8 on expression of CCR2 and CXCR4 was also investigated. Stimulation with rh-CXCL8 increased CCR2 mRNA expression in both castrate-resistant cell lines, irrespective of their PTEN status (Fig 1C, left panel), and in each of the androgen-sensitive lines, LNCaP and 22Rv1 (Fig 1C, left panel). Similarly, rh-CXCL8 signaling increased CXCR4 mRNA levels in all four cell lines relative to un-stimulated controls (Fig 1C, right panel). Furthermore, QPCR analysis demonstrated that the magnitude of increased CCR2 and CXCR4 mRNA expression was similar in the isogenic DU145 11.02 and NT.01 clones, supporting that CXCL8-induced regulation of these chemokine receptors is independent of PTEN expression (Fig S1B). We also determined that CXCL8 signaling increased CXCR7 mRNA expression, a further receptor shown to mediate the biological activity of CXCL12 (Fig S1C). QPCR data was confirmed by immunoblotting analysis, which confirmed CXCL8-induced up-regulation of CXCR4 receptor protein expression in PC3 and LNCaP cells (Fig 1B). Therefore, our data indicates that CXCL8 signaling originating from PTEN-deficient tumor cells increases the expression of CCR2 and CXCR4 in prostate epithelium, but of potential significance, we observed that the expression of these receptors can be induced in prostate epithelial cells that still retain functional PTEN and have the capacity to respond to CXCL8.

### CXCL8 signaling regulates CCL2 and CXCL12 expression and secretion in prostate stromal cells

**Figure 2:**
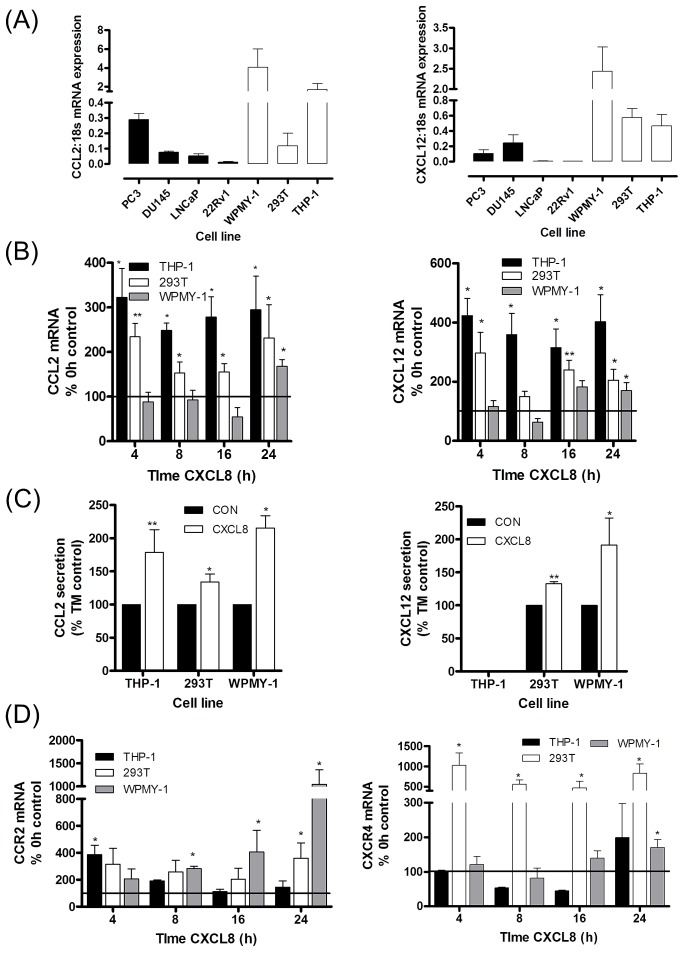
Paracrine CXCL8 signaling induces CCL2 and CXCL12 synthesis and secretion in stromal cells **(A)** Bar graph illustrating qPCR validation of CCL2 (left panel) and CXCL12 (right panel) gene expression in multiple prostate cancer and stromal cell lines. **(B)** Bar graph illustrating qPCR validation of CCL2 (left panel) and CXCL12 (right panel) gene expression in THP-1 macrophage-like cells, 293T and WPMY-1 stromal fibroblasts, subjected to stimulation with 3nM rh-CXCL8. **(C)** Bar graph illustrating the levels of CCL2 (left panel) and CXCL12 (right panel) secreted by stromal cells, in the absence and presence of stimulation with 3nM rh-CXCL8. Data shown represents the mean ± S.E.M. value determined by repetitive ELISAs. **(D)** Bar graph illustrating qPCR validation of CCR2 (left panel) and CXCR4 (right panel) gene expression in stromal THP-1 cells, 293T cells and WPMY-1 cells subjected to stimulation with 3nM rh-CXCL8. Data shown in (A), (B) and (D) is the mean ± S.E.M value, determined from a minimum of 4 replicate experiments. Statically significant differences in expression were determined by performing a two-tailed Mann-Whitney U-test (*p<0.05; ** p<0.01; ***p<0.001).

Further experiments focused on characterizing the regulation of CXCL12 and CCL2 across our panel of prostate cancer cells and representative stromal cell lines. Basal mRNA expression for CCL2 (Fig 2A, left panel) and CXCL12 (Fig 2A, right panel) was lower in each of the prostate cancer cell lines relative to the expression detected in the WPMY-1 prostate stromal fibroblast or the monocytic THP-1 cell lines. Furthermore, qPCR analysis revealed no change in CXCL12 (Fig S2A) or CCL2 (Fig S2B) mRNA expression following stimulation with rh-CXCL8 in any of the prostate cancer cell lines studied; moreover, we observed that the secretion of either CCL2 or CXCL12 from any of the prostate cancer cells was below the limits of detection of the ELISA assays employed. However, basal secretion of these chemokines from the representative stromal cell lines was easily detectable (Fig S2C).

Expression of CXCR1 and CXCR2 was also detected in the stromal cell lines and this was up-regulated in response to CXCL8 stimulation, demonstrating their responsiveness to CXCL8 stimulation (Fig S2D). Moreover, we observed a significant increase in CCL2 mRNA expression (Fig 2B, left panel) and secretion (Fig 2C, left panel) from macrophage-like THP-1 cells as well as WPMY-1 and 293T cells (both fibroblast-like cell lines) following exposure to rh-CXCL8. Increased CXCL12 mRNA expression was detected to varying degrees in CXCL8-stimulated THP-1, 293T and WPMY-1 cells (Fig 2B, left panel). CXCL12-secretion was significantly increased from both fibroblast-like cell lines including the prostatic fibroblast WPMY-1 but was undetectable from THP-1 cells (Fig 2C, right panel).

Basal expression of CCR2 and CXCR4 was also detected in each of the stromal cell lines (Fig S2E). Given the effect of CXCL8 in regulating expression and secretion of CCL2 and CXCL12 from these stromal cells, we also extended our analysis and confirmed that CCR2 (Fig 2D, left panel) and CXCR4 (Fig 2D, right panel) mRNA expression by each of these stromal cell lines was also up-regulated in response to CXCL8 stimulation.

Our initial data suggests that stromal rather than prostate cancer cells are the principal source of CCL2 and CXCL12. Moreover, the release of CXCL8 from PTEN-depleted prostate cancer cells may act in a paracrine manner to increase the expression and secretion of CCL2 and CXCL12 from neighboring stromal cells. In contrast, autocrine CXCL8 signaling increases the expression of CCR2 and CXCR12 in prostate epithelium but does not appear to affect expression of the chemokine ligands. Collectively, this data indicates that CXCL8 signaling may initiate signaling that results in increasing the sensitivity of prostate epithelium to stromal-derived chemokine signals.

### CXCL8, CCL2 and CXCL12 play differential roles in potentiating the chemotactic migration of prostate cancer cells

Local tumor migration and invasion plays an important role in metastasis and therapeutic relapse. To explore the significance of CXCL8-promoted and stromal-derived chemokine signaling on prostate cancer cell-migration, we established a series of *in vitro* experiments to characterize the functional importance of CXCL8, CXCL12 and CCL2 as independent and co-dependent migratory factors within the prostate tumor microenvironment.

**Figure 3:**
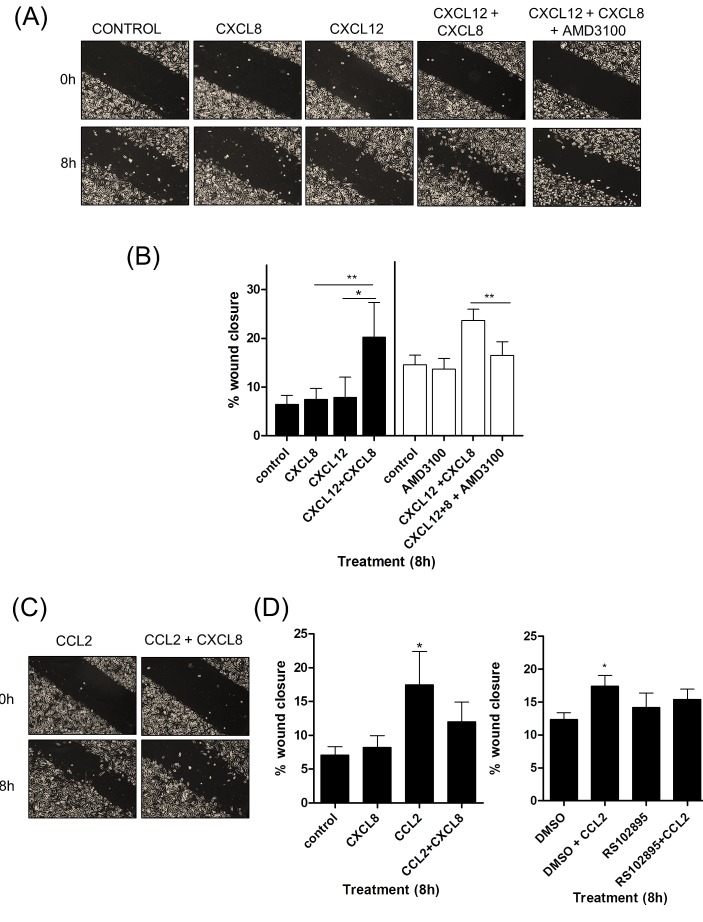
CXCL12 signaling potentiates the chemotactic migration of PC3 cells **(A)** Representative images of wound scratch assays conducted using PC3 monolayers, subjected to treatment with relevant concentrations of CXCL8 and CXCL12, or treatment with the CXCR4 inhibitor AMD3100. Images shown depict the uniformity of the wound scratch at time of initiation (t=0) and the resulting closure of the wound after 8h stimulation. **(B)** Bar graph presenting the quantitation of wound closure of a PC3 monolayer resulting from various chemokine treatments. Data shown is the mean ± S.E.M. value of three independent experiments, each performed in triplicate. **(C)** Representative images of wound scratch assays conducted using PC3 monolayers, subjected to treatment with relevant concentrations of CXCL8 and CCL2. Images shown depict the uniformity of the wound scratch at time of initiation (t=0) and the resulting closure of the wound after 6 h stimulation. **(D)** Bar graphs illustrating the extent of wound closure of the PC3 monolayers promoted by stimulation with CXCL8 or CCL2, in isolation or in combination (left panel), and the impact of administering a CCR2 antagonist RS102895 upon CCL2-induced wound closure (right panel). Data shown is the mean ± S.E.M value, determined from a minimum of 3 replicate experiments. Statistically significant differences in expression were determined by performing a two-tailed Students t-test (*p<0.05; ** p<0.01; ***p<0.001).

Using wound scratch assays, we observed no change in the migratory potential of PC3 cells when stimulated with CXCL12 (100ng/ml) or CXCL8 (3nM) alone (Fig 3A & 3B). However, a significant increase in wound closure was observed when PC3 cells were co-stimulated with CXCL8 and CXCL12. This migratory response to CXCL8 and CXCL12 was abrogated by administration of the CXCR4 antagonist AMD3100 (Fig 3A & 3B).

This co-dependent effect of CXCL8 and CXCL12 was supported in further experiments. Use of a traditional Boyden chamber experiment confirmed that migration towards a CXCL12 stimulus (lower chamber) was only observed following the addition of CXCL8 to the upper chamber of the apparatus (Fig S3, left panel). Moreover, experiments conducted over an extended timecourse on the Xcelligence platform demonstrated an increased rate of migration of CXCL8-stimulated PC3 cells towards CXCL12 (Fig S3, right panel).

In contrast, the effect of CCL2 upon PC3 cell migration was more complex. Addition of CCL2 (100ng/ml) alone significantly enhanced the rate of wound closure. However, although CCL2 and CXCL8 together increased the migratory potential of PC3 cells over control, the effect was not as dramatic as that exerted by CCL2 alone (Fig 3C & 3D, left panel). CCL2-promoted PC3 cell motility was arrested by the administration of the CCR2 antagonist RS102895 (Fig 3D).

Similar experiments were performed on isogenic DU145 clones. We observed a significant increase in wound closure when PTEN-expressing DU145 NT.01 and PTEN-depleted DU145 11.02 cells were cultured with CXCL8 in combination with CXCL12(Fig S4), eliminating the potential that this response was specific to PC3 cells. Thus, our experiments indicate that CXCL8 and CXCL12 signaling co-operate to accelerate the migration of prostate cancer cells.

### CXCL-8-induced stromal-derived CXCL12 secretion promotes PC3 cell migration

Co-culture experiments were conducted to examine whether CXCL8-induced increases in stromal cell-derived CXCL12 expression could potentiate prostate cancer cell migration. CXCL8-secreting PC3 cells were co-cultured overnight with either THP-1 or WPMY-1 cells prior to wound scratch assays, allowing signaling between tumor and stromal cells to be established.

**Figure 4:**
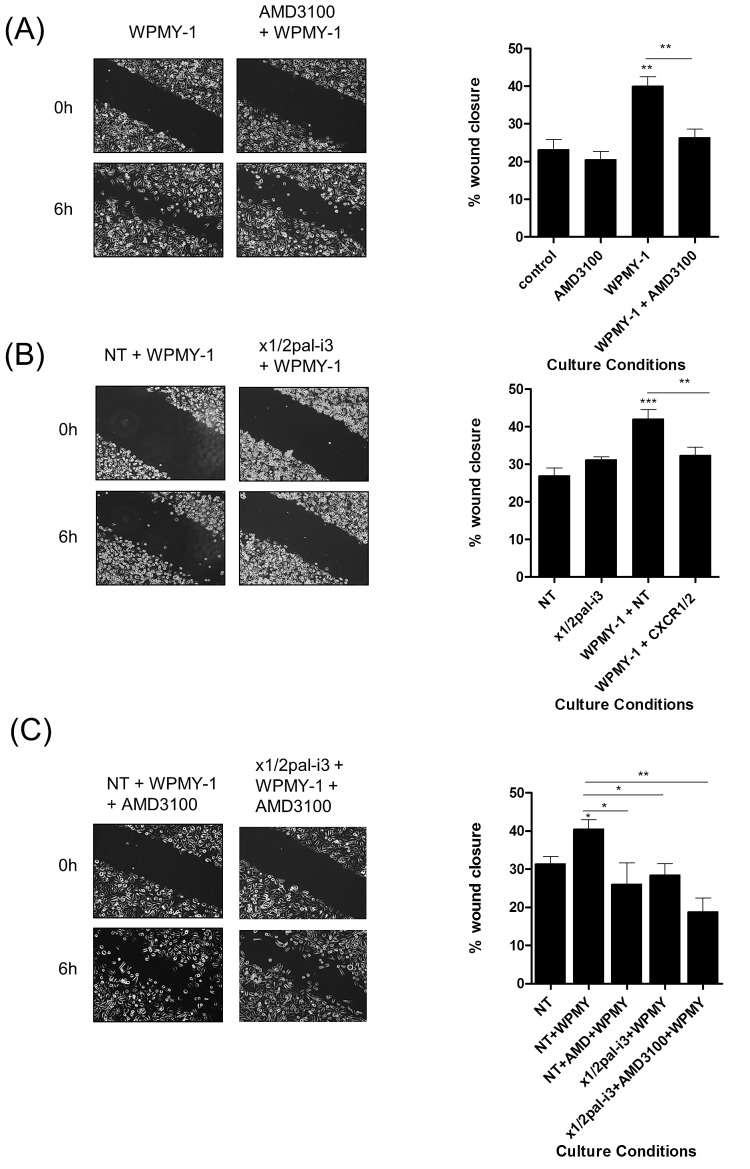
Fibroblasts accelerate PC3 cell motility through a CXCR4-dependent mechanism **(A)** *Left;* Representative images of wound scratch assays conducted using PC3 monolayers in the presence of WPMY-1 prostate stromal fibroblasts and the impact of administering the CXCR4 antagonist AMD3100. *Right;* Bar graphs presenting the quantification of wound closure observed under various treatments in wound scratch assays. Data shown is the mean ± S.E.M. value calculated from 4 independent experiments, each performed in duplicate/triplicate. Images shown depict the uniformity of the wound scratch at time of initiation (t=0) and the resulting closure of the wound after 6h stimulation. **(B)** Representative images of wound scratch assays conducted using PC3 monolayers in the presence of WPMY-1 prostate stromal fibroblasts and the impact of administering the x1/2pal-i3 pepducin to the co-culture and corresponding bar graphs presenting the quantification of wound closure. Data shown is the mean ± S.E.M. value calculated from 4 independent experiments, each performed in duplicate/triplicate. **(C)** Representative images of wound scratch assays conducted using PC3 monolayers in the presence of WPMY-1 prostate stromal fibroblasts and corresponding bar graphs presenting the quantification of wound closure. Data shown is the mean ± S.E.M. value calculated from 4 independent experiments, each performed in duplicate/triplicate. Images show the effect of administering the CXCR4 inhibitor AMD3100 in the absence and presence of the CXCR1/CXCR2-targeted x1/2pal-i3pepducin upon the WPMY-1 fibroblast accelerated wound closure of PC3 cells. Statistically significant differences in expression were determined by performing a two-tailed Students t-test (*p<0.05; ** p<0.01; ***p<0.001).

WPMY-1 cells acted to promote the migration of PC3 cells (Fig 4A). Addition of the CXCR4-selective antagonist AMD3100 significantly attenuated this response, confirming the importance of CXCL12/CXCR4 signaling in enabling this fibroblast-enhanced migration. Similarly, inhibition of CXCL8 signaling using a peptide-based receptor-targeting pepducin (x1/2pal-i3) [22] was also shown to significantly attenuate the WPMY-1-induced migration of PC3 cells (Fig 4B). In a further series of parallel experiments, we observed that while targeting either the tumor-derived signaling stimulus, CXCL8 (using x1/2pal-i3), or the fibroblast-derived enabler of migration, CXCL12 (using AMD3100), attenuated the migration of PC3 cells to similar extents, a trend towards more effective attenuation of WPMY-1-promoted wound closure was observed following the simultaneous administration of both AMD3100 and x1/2pal-i3 (Fig 4C).

**Figure 5:**
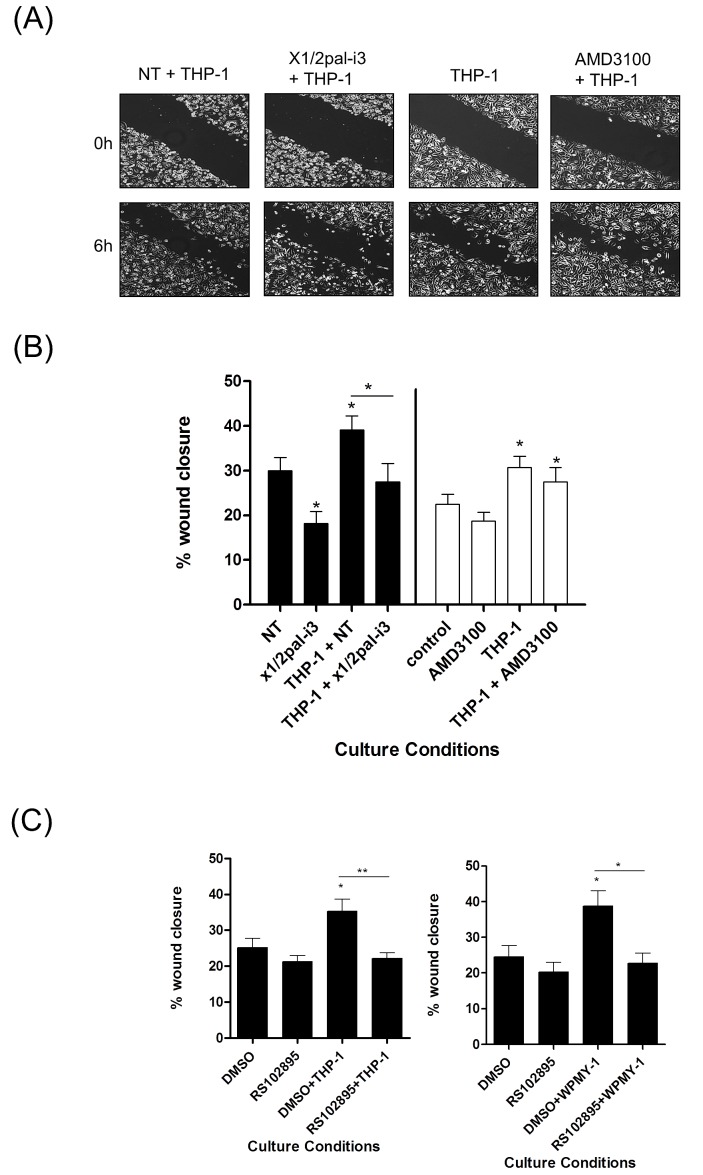
Macrophage-promoted acceleration of prostate cancer cell motility is sensitive to CXCR1/CXCR2 inhibition **(A)** Representative images of wound scratch assays conducted using PC3 monolayers, examining the impact of THP-1 cells upon the acceleration of prostate cancer cell motility, in the absence and presence of the CXCR1/CXCR2-targeting x1/2i3-pal and the CXCR4 inhibitor AMD3100. Images shown depict the uniformity of the wound scratch at time of initiation (t=0) and the resulting closure of the wound after 6 h stimulation. **(B)** Bar graph presenting the quantification of wound closure effected by the addition of THP-1 cells to PC3 cell monolayers and the impact of administering the CXCR1/CXCR2-targeting x1/2pal-i3pepducin or the CXCR4 antagonist AMD3100 to the co-culture. Data shown is the mean ± S.E.M. value calculated from 4 independent experiments, each performed in duplicate/triplicate. **(C)** Bar graphs presenting the quantification of wound scratch assays examining the effect of the CCR2 inhibitor RS102895 on THP-1 (left panel) or WPMY-1 (right panel)-induced migration of PC3 cells. Data shown is the mean ± S.E.M. value calculated from 4 independent experiments, each performed in duplicate/triplicate. Statistically significant differences in expression were determined by performing a two-tailed Students t-test (*p<0.05; ** p<0.01; ***p<0.001).

Addition of THP-1 cells also promoted PC3 wound closure (Fig 5A/B). Interestingly, this response was inhibited by addition of x1/2pal-i3 pepducin while administration of AMD3100 had no effect, consistent with our observation that THP-1 cells do not secrete detectable levels of CXCL12. Addition of the CCR2 inhibitor RS102895 attenuated both THP-1- and WPMY-1-promoted migration of PC3 cells (Fig 5C), consistent with the secretion of CCL2 from each of these stromal cells.

### CXCL8, CXCL12 and CCL2 have differential impacts on the proliferation and survival of prostate cancer cells

We investigated the role of CXCL8, CCL2 and CXCL12 in regulating the growth and survival of prostate cancer cells, independently or in a co-dependent manner. Stimulation of PC3 cells with CCL2 resulted in a concentration-dependent increase in the proliferation rate. In contrast, stimulation with increasing concentrations of CXCL12 inhibited the proliferation rate of PC3 cells (Fig S5A). Accordingly, further experiments investigated the importance of stromal-derived CCL2 signaling on prostate cancer cell proliferation/viability.

**Figure 6:**
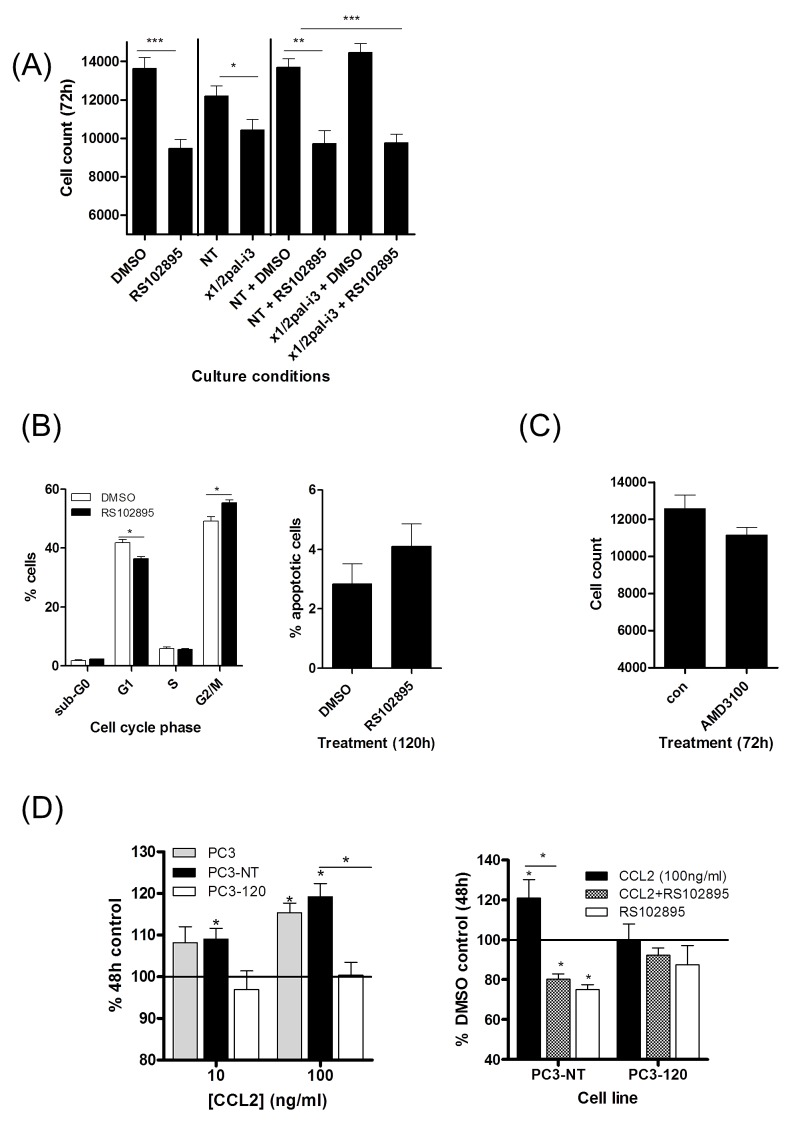
Characterization of the effects of CCL2 and CXCL12 on the proliferation and viability of prostate cancer cells **(A)** Bar graph presenting cell count data illustrating the effect of administering the CXCR1/CXCR2-targeting inhibitor x1/2pal-i3or the CCR2 antagonist RS102895, independently or in combination upon the proliferation of PTEN-deficient PC3 cells. **(B)** Flow cytometry data demonstrating the effect of administering RS102895 upon cell cycle progression (left panel) and cell viability (right panel). **(C)** Bar graph presenting cell count data illustrating the effect of the CXCR4 inhibitor AMD3100 on the proliferation of PC3 cells. **(D)** Left panel; Bar graph presenting the effect of CCL2 (100ng/mL) upon the viability of PC3 cells, transfection-control PC3 cells (PC3-NT) or low CXCL8-expressing PC3 cells, assessed by MTT assay. Right panel; bar graph illustrating the effect on cell proliferation affected by administering the CCR2 antagonist RS102895 to PC3-NT or low CXCL8-expressing PC3-120 cells in the absence and presence of CCL2 (100 ng/mL). Statistically significant differences in expression were determined by performing a two-tailed Students t-test or two-tailed Mann-Whitney U test (*p<0.05; ** p<0.01; ***p<0.001).

Treatment with the CCR2 antagonist RS102895 significantly reduced the viability of PC3 cells in both a cell count assay (Fig 6A) and a MTT assay (Fig S5B). Cell cycle analysis demonstrated a significant increase in the percentage of cells in the G2/M phase of the cell cycle following administration of RS102895 (Fig 6B, left panel). However, inhibition of CCL2 signaling using this antagonist did not increase the level of apoptotic cells detected by PI/annexin V-labelling (Fig 6B, right panel). Consistent with our published reports, the inhibition of CXCL8 signaling using x1/2pal-i3 similarly reduced the viability of PTEN-depleted PC3 cells (Fig 6A & Fig S5B). However, we observed no additional benefit from co-administering RS102895 and x1/2pal-i3 in the context of reducing cell viability (Fig 6A). As further internal validation of our data, treatment with the CXCR4 inhibitor AMD3100 had no effect on basal cell number relative to untreated controls or in affecting the viability of the PC3 cells in MTT assays (Fig 6C & Fig S5B).

### Autocrine CXCL8 signaling augments sensitivity of PC3 cells to CCL2 stimulation

Elevated CXCL8 expression and the characterization of autocrine signaling effects of CXCL8 in PC3 cells is well-documented [20,23]. We developed further derivative PC3 models to investigate how the levels of autocrine CXCL8 signaling may augment the ability of CCL2 to enhance cell proliferation. PC3 cells were transfected with a CXCL8-targeted shRNA. While transfection protocols with shRNA did result in the loss of PC3 viability, we were able to select a small number of residual populations; CXCL8 expression within the selected populations was characterized by qPCR analysis and ELISA. For the purpose of this study, a population with CXCL8 expression equivalent to parental PC3 cells (PC3-NT) and a population in which CXCL8 expression was repressed by ~25% relative to PC3-NT levels (PC3-120; Fig S5C) were used in further experimentation. Consistent with our earlier results indicating that CXCL8 signaling up-regulated CCR2 and CXCR4 expression in prostate cancer cells, we observed that the reduction in autocrine CXCL8 expression resulted in a significant decrease in CCR2 and CXCR4 mRNA expression in PC3-120 cells (Fig S5C). Growth curve analysis demonstrated that there was no significant difference in the basal growth rate observed between parental PC3, PC3-NT and PC3-120 cells (Fig S5D). Stimulation of PC3-NT cells with rh-CCL2 promoted cell proliferation to a similar extent as observed in parental PC3 cells (Fig 6D, left panel). In contrast, CCL2 had no effect on growth of the low CXCL8-expressing PC3-120 cells (Fig 6D, left panel). Addition of RS012895 attenuated CCL2-promoted growth of PC3-NT cells, but there was no effect on the proliferation of PC3-120 cells (Fig 6D, right panel).

## DISCUSSION

*PTEN-*deficiency is a major molecular hallmark of prostate cancer [4]. We have associated increased expression and secretion of the pro-inflammatory CXC-chemokine CXCL8 with loss of *PTEN* function in prostate cancer cells and prostatic tissue [20]. Furthermore, loss of *PTEN* function concurrently increased the expression of CXCL8 receptors (CXCR1 and CXCR2), with the resulting elevated autocrine CXCL8 signaling acting to sustain the viability of these *PTEN*-deficient prostate cancer cells [20]. Beside their elevated expression in *PTEN*-deficient prostate cancer cells, CXCR1 and CXCR2 are also expressed on vascular endothelial cells, monocytes and fibroblasts [24,25]. Therefore, each of these cell types within the tumor microenvironment is potentially receptive to the increased levels of CXCL8 secreted by *PTEN*-deficient prostate cancer cells. The objective of this study was to characterize the functional importance, and molecular basis, of CXCL8-promoted tumor-stromal communication in promoting the aggressiveness of PTEN-deficient prostate cancer cells, with a specific focus on understanding how tumor-derived CXCL8 may regulate the expression of stromal-derived chemokines.

Our data highlights a co-ordinated response of tumor and stromal cells to the secretion of CXCL8 from malignant prostate cancer cells. Autocrine CXCL8 signaling was shown to-upregulate the expression of chemokine receptors in prostate cancer cells. Interestingly, CXCL8 signaling increased the expression of CXCR1 and CXCR2 receptors in *PTEN*-deficient prostate cancer cells, suggesting that CXCL8 signaling acts in a self-sustaining capacity, to potentiate its signaling in *PTEN*-deficient prostate cancer. Furthermore, CXCL8 signaling also increased the expression of CXCR4 and CXCR7 (the receptors mediating the biological activity of CXCL12) and CCR2 (a receptor activated by the ligand CCL2) in both PTEN-expressing and PTEN-deficient prostate cancer cells.

While CXCL8 was unable to induce chemokine synthesis in prostate cancer cells, CXCL8 signaling did increase CXCL12 and CCL2 synthesis in prostate stromal fibroblasts and monocytes. Elevated CXCL12 secretion was observed from cells of fibroblast origin while increased CCL2 secretion was demonstrated in fibroblasts and monocytes. CXCL8 signaling also increased CXCR4 and CCR2 expression in these stromal cells. Although we have not investigated the significance of these concurrent increases in chemokine ligand and receptor expression upon the function of the stromal cells themselves, it is conceivable that this elevated chemokine signaling may regulate stromal cell function. For example, CCL2 is known to drive the differentiation of macrophages towards the M2 phenotype [26], typically associated with a tumor progression. Moreover, CCL2 can drive the infiltration of lymphocyte populations [27]. We propose that, in addition to immediate impacts on tumor cells, tumor-derived CXCL8 may also initiate sustained, long-term effects on the tumor microenvironment, by activating resident stromal cells, which, in turn, may act to either directly remodel the tumor site or promote the recruitment of additional cell types. Previous studies have also demonstrated the importance of fibroblast-derived CXCL12 in the evasion of immunosurveillance in pancreatic ductal adenocarcinoma [28]. Our current findings propose that tumor-derived CXCL8, through the regulation of fibroblast-derived CXCL12 production, may drive immune evasion in prostate cancer.

**Figure 7:**
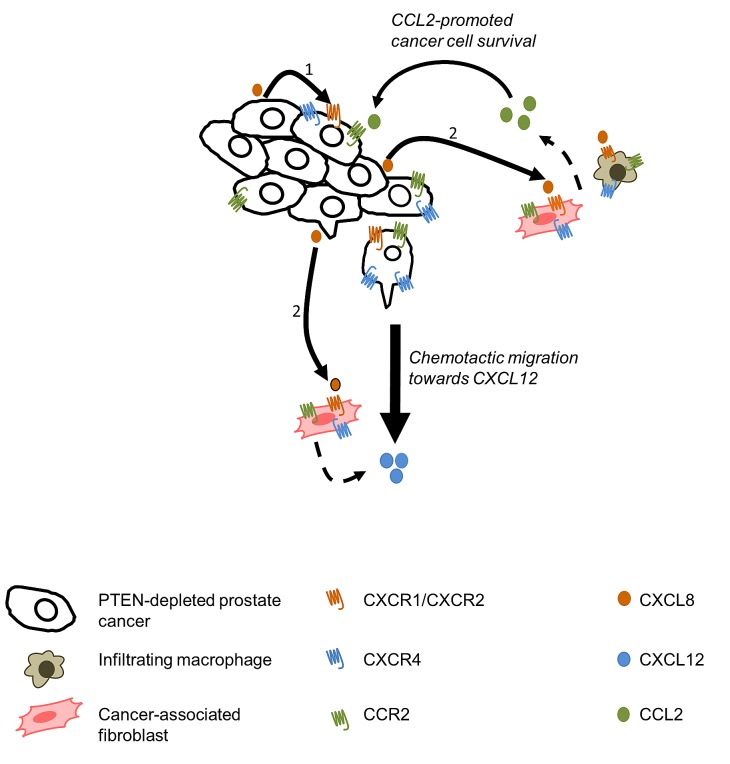
Schematic diagram representing chemokine crosstalk within the tumor microenvironment 1. Autocrine CXCL8 signaling results in the up-regulation of CXCR1, CXCR2, CXCR4 and CCL2 on the tumor cells. 2. Paracrine CXCL8 signaling leading to the up-regulation of CXCR1, CXCR2, CXCR4 and CCR2 expression by tumor-associated stromal cells. Paracrine CXCL8 signaling also induces secretion of CCL2 by macrophages and fibroblasts, as well as CXCL12 secretion by tumor-associated macrophages.

CXCL8 did not promote migration of prostate cancer cells; elevated secretion of CXCL8 by prostate tumors results in locally high levels of this chemokine which in turn may prevent the establishment of a chemotactic CXCL8 gradient. Instead, integrating our observations in tumor and stromal cell lines, we propose that tumor-derived CXCL8 signaling initiates a co-ordinated chemokine-driven cross-talk between tumor and stromal cells (Fig 7). Our data demonstrates that tumor-derived CXCL8 acts to increase the secretion of stromal-derived chemokines which then activate the increased pool of their respective receptors expressed on the surface of prostate cancer cells. Ultimately, the combined effect is that CXCL8 signaling increases the sensitivity of prostate cancer cells to stromal-derived chemokines. Our functional experiments establish that CXCL12 and CCL2 act upon prostate cancer cells to induce very different responses. While CXCL12 was clearly implicated in supporting fibroblast-dependent migration of PC3 cells, CCL2 signaling was observed to be more effective in underpinning monocyte-dependent proliferation of the malignant cells. For either of these phenotypes, we observed that the efficacy of the stromal-derived chemokine required the presence of tumor-derived CXCL8 signaling (Fig 7). For example, in migration assays, addition of both CXCL8 and CXCL12 was necessary in order to observe migration. Furthermore, PC3 cells expressing lower levels of CXCL8 were insensitive to the proliferation-promoting effects of CCL2. We propose these results indicate the importance of CXCL8-driven CXCR4 and CCR2 expression in the prostate cancer cells, which increases their capacity to respond to each of these stromal-derived chemokines.

Addition of fibroblasts and macrophages promoted the migration and invasion of PC3 cells. CXCL12/CXCR4 signaling was shown to support fibroblast-mediated migration and invasion of PC3 cells in co-culture assays but not that promoted by macrophage-like cells. Moreover, combined inhibition of both CXCL8 and CXCL12 signaling was more effective in repressing fibroblast-promoted cell motility. In contrast, although CCL2 signaling had only a minor effect on macrophage-induced prostate cancer cell motility, this chemokine had significant effects upon the proliferation and survival of PTEN-deficient prostate cancer cells. Furthermore, repression of CXCL8 signaling reduced the sensitivity of prostate cancer cells to these effects of CCL2. These observations have important considerations in the context of chemokine signaling as therapeutic interventions. Specifically, our data suggests that targeting of only one chemokine signaling axis may not be sufficient to adequately suppress independent functional responses. Furthermore, given the interplay between tumor-derived and stromal-derived chemokines, we propose that co-targeting of multiple chemokine pathways may be required in order to diminish multiple behavioral traits of aggressive PTEN-deficient prostate cancer cells. Therefore, combined targeting of CXCR1/CXCR2, CXCR4 and CCR2 signaling may be necessary in order to deliver concurrent inhibition of stroma-promoted invasion and proliferation.

In summary, we report the characterization of a CXCL8-driven augmentation of CXCL12 and CCL2 signaling arising from different compartments of the tumor-associated stroma, which act to sustain the hallmarks of increased cell motility (migration and invasion) and increased cell proliferation and survival. Our study complements prior studies which have reported the significance of stromal-derived CXCL12 signaling in driving the invasion and metastasis of prostate cancers [14,29], and provides a more-detailed understanding of how stromal-derived chemokine signaling may be induced. We propose this chemokine cross-talk has greatest relevance to foci of PTEN-deficient prostate cancers which secrete higher levels of CXCL8 in order to trigger the induction of stromal chemokine synthesis. Dedicated studies employing immune-competent transgenic models of PTEN-deficient prostate cancer are underway in our laboratory to provide greater insight into the potential consequences of this chemokine signaling cross-talk in driving the aggressiveness of PTEN-deficient prostate carcinomas.

## MATERIALS AND METHODS

### Chemical and Reagents

Chemicals were source from Sigma-Aldrich (St. Louis, MO) unless otherwise stated.

### Cell Culture and Treatments

Prostate cancer cell lines (PC3, DU145, LNCaP and 22Rv1) or clones were sourced, derived and cultured as previously described [20]. Fibroblast (293T, human embryonic kidney; WPMY-1, prostate stroma) cell lines were maintained in DMEM (GE Healthcare, Buckinghamshire, UK) supplemented with 10% (v/v) fetal calf serum (FCS; GIBCO BRL/Life Technologies, Paisley, Scotland). Human macrophage-like THP-1 cells were maintained in RPMI supplemented with 10% FCS and 0.05M β-mercaptoethanol. All cell lines were maintained at 37°C.

In experiments investigating CXC-chemokine signaling, cells were incubated in serum-free, phenol-red free RMPI 1640 or DMEM medium for 16h prior to exposure to 3nM recombinant human (rh)-CXCL8, 100ng/ml rh-CXCL12 or 100ng/ml rh-CCL2 (Peprotech, London, UK). For inhibition of chemokine signaling, cells were treated with RS102895 (CCR2 antagonist, 10nM; Tocris, Bristol, UK), AMD3100 (CXCR4 antagonist, 25μg/ml; Tocris) or a CXCR1/2-derived pepducin (x1/2pal-i3, 300nM; Biomatik, Wilmington, Delaware). Control cells were treated with the appropriate vehicle or NT-pepducin (x1/2pal-con) sequence as required.

### Generation of PC3-120 cells

CXCL8-depleted PC3-120 and corresponding PC3-NT cells were generated by stable downregulation of CXCL8 using commercially available CXCL8-targeted shRNA and non-targeting plasmids (HuSH constructs in pGFP-V-RS vector; Origene, Rockville, MD, USA) according to the manufacturer's instructions. Briefly, PC3 cells (5 × 10^5^) were incubated in a transfection mix containing plasmid (2μg), Lipofectamine 2000 and OptiMEM medium (GIBCO BRL/Life Technologies). Following 24h, cells were reseeded and populations containing the shRNA plasmids were selected using puromycin (0.5μg/ml). PC3-NT and PC3-120 cells were maintained in RPMI 1640 medium supplemented with 10% FCS and 0.5μg/ml puromycin.

### Quantitative real time PCR (qPCR)

RNA was harvested and reverse transcribed as previously described using specified primer sequences [20]. Real-time PCR was performed in 96-well plates using an LC480 light cycler instrument (Roche Diagnostics, Burgess Hill, UK). MRNA expression levels were determined using the relative standard curve method and normalized against 18S.

### Immunoblotting

Whole cell lysates were prepared, resolved and blotted as described previously [20]. CXCR4 expression was detected using an anti-human CXCR4 antibody (Abcam) at 1:1000. Expression of CXCR1 and CXCR2 were detected as previously described [30]. Membranes were re-probed with GAPDH antibody to ensure equal loading (Biogenesis, Dorset, UK).

### ELISA

The amount of secreted CCL2 (Biolegend, London, UK) or CXCR12 (R&D systems, Abringdon, UK) detected in the medium of prostate cancer cells was determined using an ELISA assay according to the manufacturer's instructions.

### MTT Assay and Cell Count Analysis

Cells were plated and treated with chemokines or chemokine inhibitors for 48h or 72h as required. Cell number was assessed by Coulter Counter [30] while cell viability was assessed by MTT assay [31].

### Cell cycle Analysis

PI staining and cell cycle analysis was performed as previously described [30].

### Apoptosis Assay

Apoptosis was measured as previously described [20].

### Migration Assays

Wound scratch assays were performed on confluent monolayers of prostate cancer cells. Prostate cancer cells were treated and/or co-cultured with stromal cell lines as required prior to a regular scratch being made in the prostate cell monolayer. Plates were observed and imaged using Cell B software (Olympus; Southend-on-Sea, UK) incubated for the required length of time and imaged again. Wounds were measured using ImageJ software and the degree of wound closure calculated.

Migration assays were performed on uncoated transwell membranes (12μM pore size; CoStar). Cells were seeded with media alone or stimulated with media containing 3nM CXCL8 and allowed to migrate towards media alone or media containing 100ng/ml CXCL12 for 20h before the membranes were stained with crystal violet and the absorbance of each well calculated.

Cell movement through a synthetic matrix *in vitro* was assessed using the RTCA DP cell analyzer system (Roche Diagnostics, Mannheim, Germany). The upper chamber of CIM invasion plates were pre-coated with Matrigel™ (5% in SFM). Cells were stimulated with 3nM CXCL8 as required and invasion towards media alone or media containing 100ng/ml CXCL12 was investigated. Measurements were taken every 15min for 36h.

### Statistical Analysis

Two-tailed Mann-Whitney U-test or a Student's t-test was used to compare means where appropriate.

#### Supplementary Materials


