# Supplementary material for: Tumor-derived CXCL8 signaling augments stroma-derived CCL2-promoted proliferation and CXCL12-mediated invasion of PTEN-deficient prostate cancer cells
Source: Oncotarget. 2014 Jun 1;5(13):4895–908. doi: 10.18632/oncotarget.2052 (PMC4148108; doi:10.18632/oncotarget.2052)
Supplement: Supplementary file 1 [file oncotarget-05-4895-s001.pdf]

Tumor-derived CXCL8 signaling augments stroma-derived CCL2-promoted proliferation and CXCL12-mediated invasion of PTEN-deficient prostate cancer cells

Supplementary Material

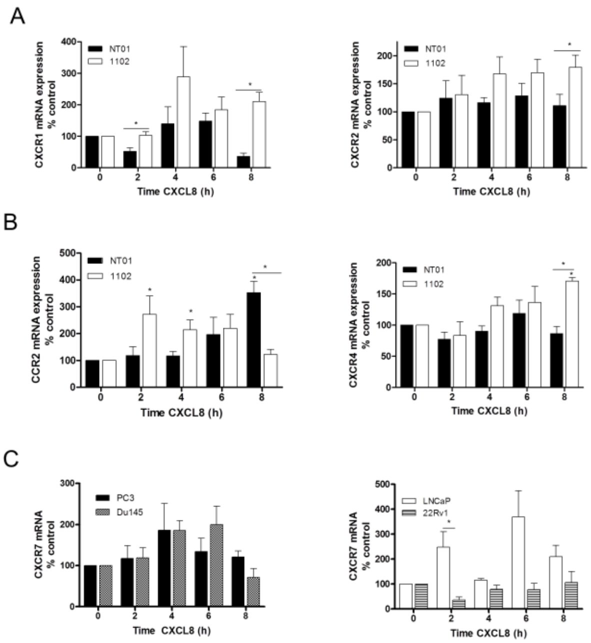

Figure S1: Effect of CXCL8 on chemokine receptor expression by prostate cancer cells. (A) Bar graph illustrating qPCR validation of CXCR1 (left panel) and CXCR2 (right panel) gene expression in PTEN-expressing (NT.01) and PTEN-depleted (11.02) DU145 cells, subjected to stimulation with 3nM rh-CXCL8. **(B)** Bar graph illustrating qPCR validation of CCR2 (left panel) and CXCR4 (right panel) gene expression in PTEN-expressing (NT.01) and PTEN-depleted (11.02) DU145 cells, subjected to stimulation with 3nM rh-CXCL8. **(C)** Bar graph illustrating qPCR validation of CXCR7 gene expression in multiple prostate cancer cell lines, following stimulation with 3nM rh-CXCL8. Data shown is the mean ± S.E.M value, determined from a minimum of 4 replicate experiments. Statistically significant differences in expression were determined by performing a two-tailed Students t-test or two-tailed Mann-Whitney U-test (*p<0.05; ** p<0.01; ***p<0.001).

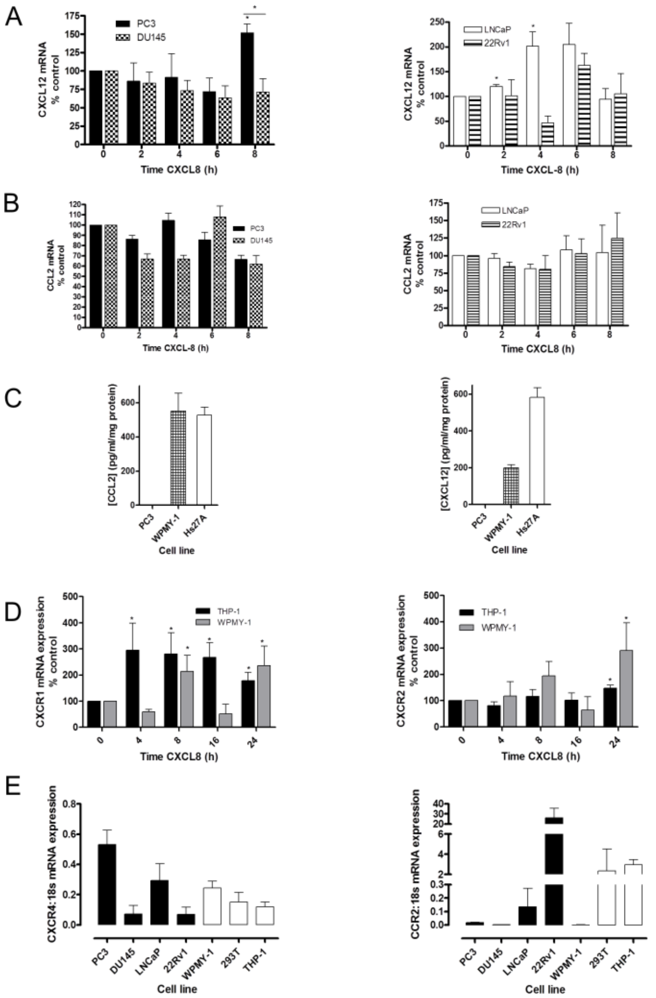

Figure S2: Effect of CXCL8 on expression of CCL2, CXCL12 and basal receptor expression. (A) Bar graph illustrating qPCR validation of CXCL12 gene expression in multiple prostate cancer cell lines, following stimulation with 3nM rh-CXCL8. **(B)** Bar graph illustrating qPCR validation of CCL2 gene expression in multiple prostate cancer cell lines, subject to stimulation with 3nM rh-CXCL8. Data shown is the mean ± S.E.M value, determined from a minimum of 4 replicate experiments. **(C)** Bar graph illustrating the basal levels of CCL2 (left panel) and CXCL12 (right panel) secreted by stromal cells, as determined by specific ELISAs. Value shown represent the mean ± S.E.M. determined by repetitive ELISAs. **(D)** Bar graphs illustrating qPCR data demonstrating the relative expression of CXCR1 (left panel) and CXCR2 (right panel) in THP-1 and WPMY-1 cells following stimulation with 3nM rh-CXCL8. Data shown is the mean **(E)** Bar graph illustrating the relative expression of CCR2 (left panel) and CXCR4 (right panel) in unstimulated prostate cancer cell lines (black bars) and stromal cell lines (open bars). Values shown are the mean ± S.E.M value, determined from a minimum of 4 replicate experiments. Statistically significant differences in gene expression were determined by performing a two-tailed Students t-test or two-tailed Mann-Whitney U-test (*p<0.05; ** p<0.01; ***p<0.001).

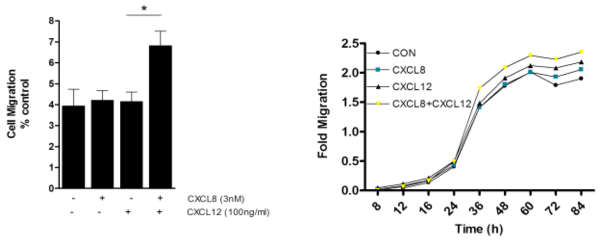

Figure S3. Effect of CXCL12 or CCL2 signaling on the chemotactic migration of PC3 cells. Left panel; Bar graph presenting data obtained from Boyden chamber migration assays, demonstrating the effect of CXCL8 (upper chamber) and CXCL12 (lower chamber) on migration of PC3 cells following 16h. Right panel; Line graph illustrating data obtained from Xcelligence migration assays, demonstrating the effect of CXCL8 and CXCL12 on migration of PC3 cells over an 84h timecourse. Data shown is the mean ± S.E.M value, determined from a minimum of 3 replicate experiments. Statistically significant differences in expression were determined by performing a two-tailed Students t-test (*$p<0.05$).

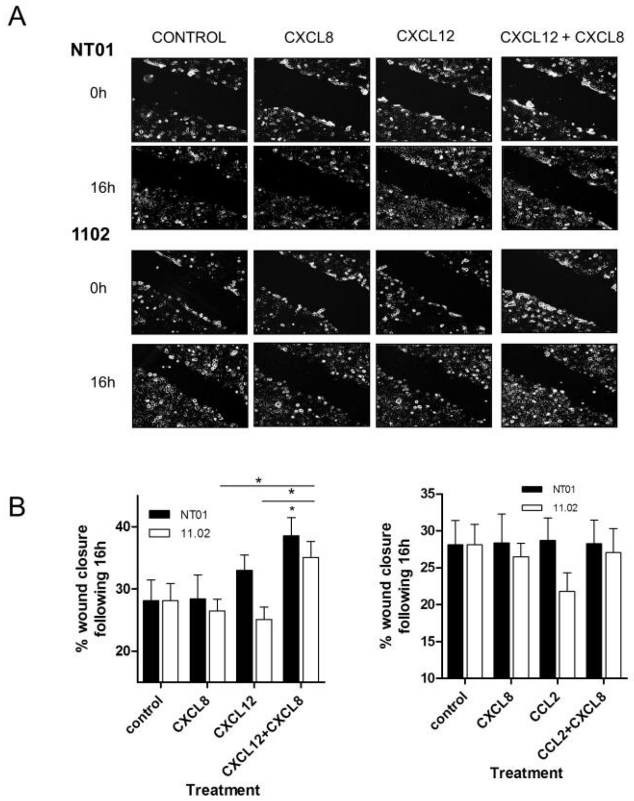

Figure S4: CXCL12 signaling potentiates the chemotactic migration of DU145 cells. (A) Representative images of wound scratch assays conducted using DU145-NT.01 or DU145-11.02 monolayers, subjected to treatment with relevant concentrations of CXCL8 and CXCL12. Images shown depict the uniformity of the wound scratch at time of initiation (t=0) and the resulting closure of the wound after 16h stimulation. **(B)** Bar graph illustrating the extent of wound closure of DU145-NT.01 or DU145-11.02 monolayers resulting from various chemokine treatments. Data shown is the mean ± S.E.M. value of three independent experiments, each performed in triplicate. Statistically significant differences in expression were determined by performing a two-tailed Students t-test (* $p<0.05$; ** $p<0.01$; *** $p<0.001$).

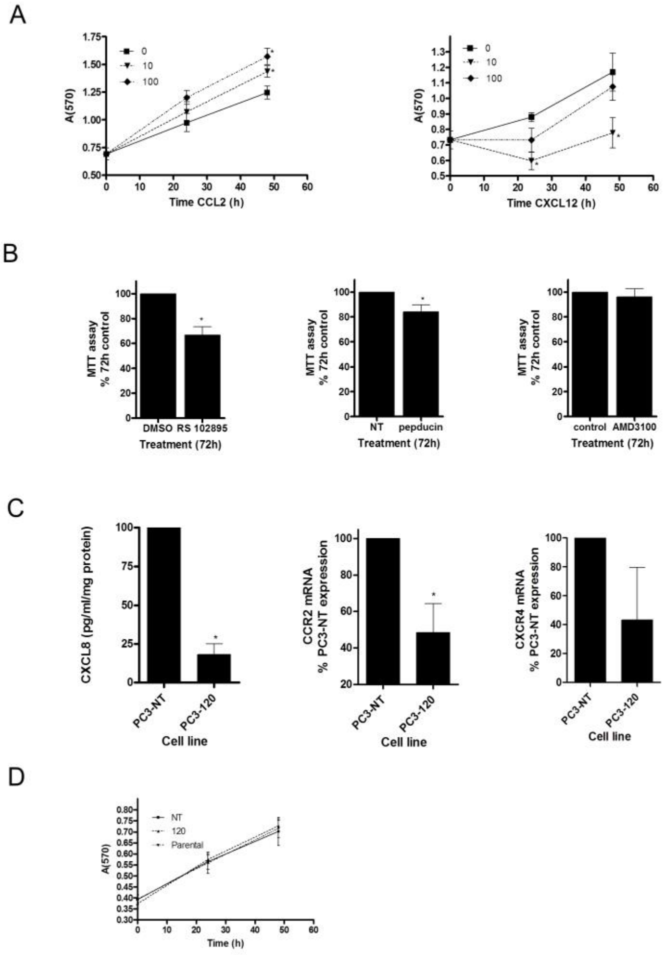

Figure S5: Characterization of the effects of CCL2 and CXCL12 on the proliferation and viability of prostate cancer cells. (A) Line graphs presenting the effect on the viability of PTEN-deficient PC3 prostate cancer cells of administering increasing concentrations of CCL2 (left panel) and CXCL12 (right panel). **(B)** Bar graph presenting MTT data illustrating the effect of administering the CCR2 antagonist RS102895, the CXCR1/CXCR2-targeting inhibitor x1/2pal-i3 or the CXCR4 inhibitor AMD3100 upon the viability of PC3 cells. **(C)** Characterization of the PC3-120 model; Left panel; Bar graph presenting ELISA data confirming that the basal secretion of CXCL8 by the PC3-120 cell population is significantly lower than that in PC3-NT cells. Middle Panel*;* Bar graph presenting qPCR data illustrating the repression of basal CCR2 mRNA expression in a PC3-120 cell population relative to that detected in a PC3-NT cell population. Right panel*;* Bar graph presenting qPCR data illustrating the repression of basal CXCR4 mRNA expression in a PC3-120 cell population relative to that detected in a PC3-NT cell population. **(D)** Line graph comparing the basal viability and cell proliferation dynamics of parental PC3, PC3-NT and PC3-120 cell populations over 48h as determined by MTT data. Data shown is the mean ± S.E.M. value of three independent experiments. Statistically significant differences in expression were determined by performing a two-tailed Students t-test or a two-tailed Mann-Whitney U test (*p<0.05; ** p<0.01; ***p<0.001).
